# Comparing the Effects of a Short Nap and Non-Sleep Deep Rest on Perceptual, Cognitive, and Physical Performance in Active Adults

**DOI:** 10.3390/clockssleep8030052

**Published:** 2026-08-28

**Authors:** Omar Boukhris, Haresh Suppiah, Matthew Driller

**Affiliations:** Centre for Sport and Human Performance Research, School of Allied Health, Human Services, and Sport, La Trobe University, Melbourne, VIC 3086, Australia; o.boukhris@latrobe.edu.au (O.B.); h.suppiah@latrobe.edu.au (H.S.)

**Keywords:** napping, NSDR, relaxation, fatigue, human performance

## Abstract

This study compared the effects of a 25-min nap opportunity and a 10-min non-sleep deep rest (NSDR) condition on perceptual, cognitive, and physical performance in physically active young adults. Sixty participants (26 female, 34 male; 22 ± 4 years) were randomly assigned to one of three groups (nap, NSDR, control; *n* = 20 each). All groups completed identical assessments immediately, 20 min, and 40 min post-intervention. Mixed-effects models, adjusted for sex, prior-night sleep, and weekly physical activity, revealed a significant group × time interaction for sleepiness, fatigue, readiness to perform, and handgrip strength (*p* < 0.05), whereas no significant group × time interaction was found for cognitive performance (reaction time and accuracy; *p* > 0.05). At 40 min post-intervention, the nap group reported lower fatigue than control (*p* = 0.01, Cohen’s d = 0.98) and higher readiness to perform than both control (*p* < 0.001, Cohen’s d = 1.48) and NSDR (*p* = 0.001, Cohen’s d = 1.25). For handgrip strength, although a significant group × time interaction was observed, post-hoc comparisons showed no significant between-group differences at any time point. No significant effects were observed for the NSDR condition on perceptual, cognitive, or physical outcomes (*p* > 0.05). These findings indicate that a short nap can enhance perceived readiness and reduce fatigue after a brief latency period, whereas NSDR did not elicit significant effects under the present conditions.

## 1. Introduction

Insufficient sleep and poor sleep quality are common across students, professionals, and athletes, often driven by busy schedules, competition demands, academic pressures, travel, and stress [1,2]. These disturbances impair attention, memory, emotional regulation, and work performance [3], and excessive daytime sleepiness has been linked to poorer academic outcomes [4]. Comparable effects are observed in workplace and sport settings, where inadequate sleep reduces performance, disrupts mood, elevates stress, and delays recovery [5,6]. To mitigate these effects, various techniques have been explored, including daytime napping [7,8], mindfulness and meditation [9,10], and biofeedback-assisted breathing exercises [11].

Recently, non-sleep deep rest (NSDR) has emerged as an innovative recovery method involving guided relaxation without the need for sleep or extensive training [12,13]. NSDR is a contemporary, secular rest strategy that shares structural features with traditional Yoga Nidra practices, including audio guidance, a quiet resting posture, breath cues, and body-focused attention. It is typically delivered through a brief audio-guided session involving body scans (i.e., systematically shifting attention from the toes to the head), slow nasal breathing, and focused awareness on internal sensations or external sounds. However, classical Yoga Nidra is embedded within a broader yogic and contemplative framework and commonly incorporates elements such as intention setting through sankalpa, structured rotation of awareness across body regions, breath awareness that may involve counting, and longer sessions [14]. In contrast, the NSDR protocol uses a brief, standardized sequence of breathing and body-focused attentional cues without these broader contemplative components or prior practice requirements [12,13,15]. NSDR also differs from mindfulness meditation in its format and purpose, which typically emphasizes the cultivation of sustained, non-judgemental awareness of present-moment experience, whereas the NSDR protocol consists of a brief, fully guided rest sequence intended to facilitate rapid relaxation without prior attentional training. This structured guidance further distinguishes NSDR from quiet wakeful rest, which involves resting without directed breathing, body scanning, or attentional instructions. NSDR can therefore be considered a structured form of guided relaxation; however, the term NSDR in the present study refers specifically to this standardized combination of guided breathing, body scanning, and focused attention.

NSDR may facilitate a state that differs from sleep itself but appears to offer some of the same restorative benefits [12,15]. NSDR may enhance performance and mood by promoting parasympathetic dominance, resulting in reduced stress and improved autonomic balance [12,15]. Additionally, from a psychological perspective, guided relaxation and visualization techniques inherent in NSDR may reduce anxiety and enhance mood by facilitating mental detachment and promoting relaxation [16]. Previous work in 65 participants demonstrated that a 10-min NSDR condition significantly enhanced physical performance, cognitive function, and mood-related measures immediately following the intervention [12].

Despite these promising findings, direct comparisons of NSDR to napping strategies, particularly daytime napping [7,8], remain notably absent in the current literature, highlighting a critical knowledge gap. This comparison is essential because NSDR is often described as producing restorative benefits similar to sleep, but through conscious relaxation rather than actual sleep. If NSDR can replicate or even approximate the physiological and perceptual effects of a nap without inducing sleep inertia or requiring a sleep-conducive environment, it could represent a highly practical alternative for individuals with limited time or opportunities to rest. Further, for those individuals who cannot nap during the day, NSDR may be a viable alternative.

Daytime naps are widely recognized for their positive effects on recovery, cognitive function, and physical performance [7,8,17]; however, they may also present practical drawbacks, such as the requirement for a suitable environment, variable nap durations, and sleep inertia, a temporary reduction in performance and alertness following awakening, lasting up to an hour [17]. Conversely, NSDR offers potential advantages, including a shorter and more consistent duration (approximately 10 min), minimal preparation, absence of sleep inertia, and greater feasibility for individuals with demanding schedules or environments lacking conducive conditions for napping [12].

Therefore, the present study aims to directly compare the effectiveness of a 10-min NSDR condition, a 25-min nap opportunity, and a control condition on perceptual, cognitive, and physical performance outcomes in physically active individuals. Based on prior evidence that both napping and NSDR can benefit perceptual, cognitive, and physical outcomes, we hypothesized that both the nap and NSDR conditions would result in more favorable perceptual, cognitive, and physical performance outcomes relative to control. Given the shorter duration of NSDR and the absence of sleep inertia, we further hypothesized that NSDR-related benefits would be evident immediately post-intervention, whereas nap-related benefits would become more evident after a delay sufficient for sleep inertia to dissipate. Physically active adults were selected because regular training and sport participation can create fluctuations in fatigue and readiness while placing demands on both cognitive and physical performance. In addition, training schedules, competition, travel, and other daily commitments may limit opportunities for extended rest, making brief daytime strategies such as napping and NSDR particularly relevant for this population.

## 2. Results

**Figure 1:**
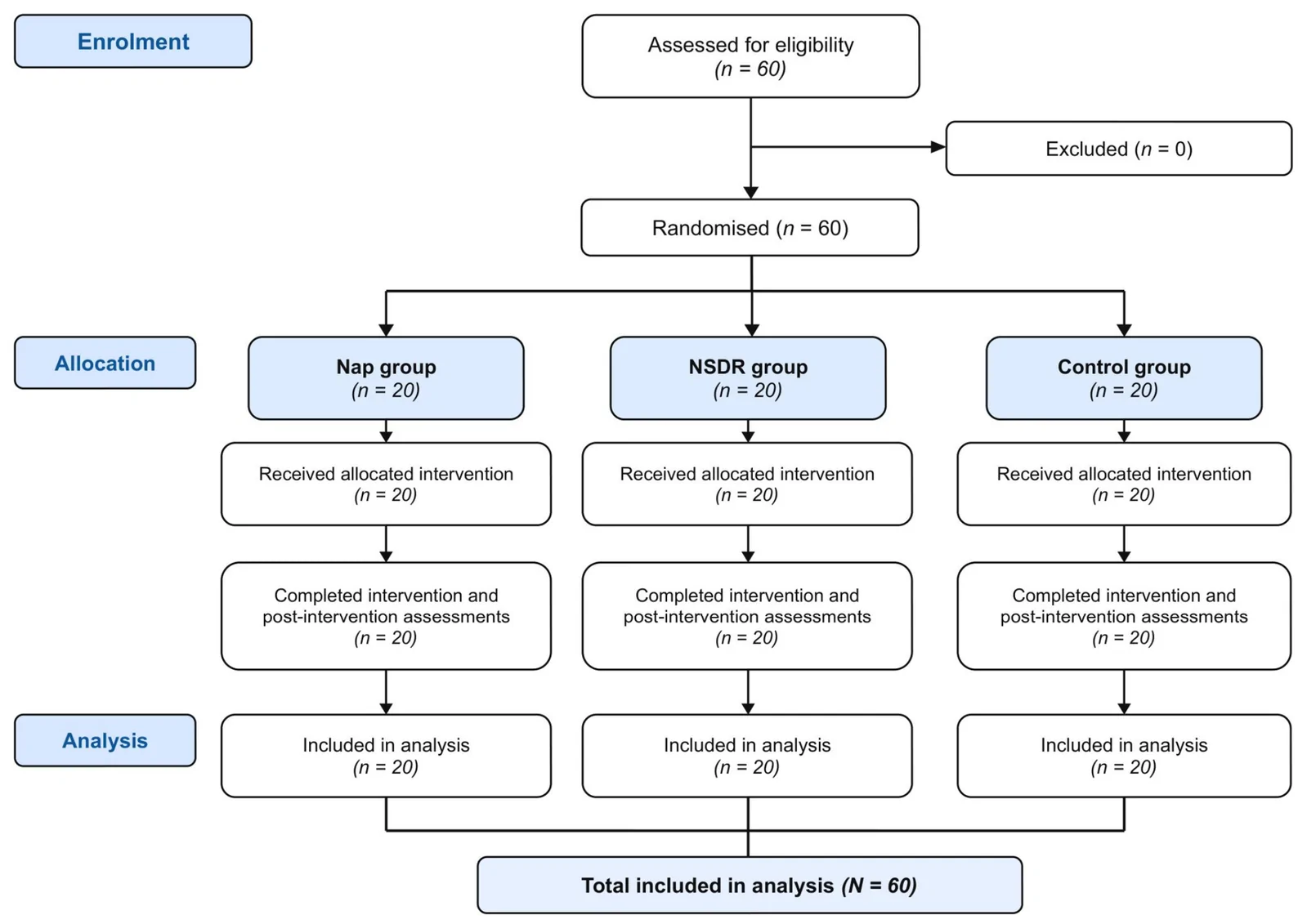
CONSORT flow diagram of participant recruitment, randomization, completion, and analysis.

Participant flow through the study is presented in Figure 1. The characteristics of participants within the subgroup samples are summarized in Table 1. Age and height differed significantly between groups (*p* = 0.003 and *p* = 0.046, respectively). However, sex distribution, body mass, physical activity, and previous night’s sleep duration did not differ significantly between groups (*p* > 0.05).

The linear mixed-effects models are presented in Table S1, and model fit indices (intraclass correlation coefficients (ICC), marginal R^2^, and conditional R^2^) are reported in Table S2.

Descriptive physiological measures recorded during the nap, NSDR, and control conditions are presented in Table S3.

During the 25-min nap opportunity, all participants included in the nap condition obtained sleep. Total sleep time was 10 ± 5 min, including 10 ± 5 min of light sleep, 0 ± 1 min of deep sleep, and 0 min of rapid eye movement (REM) sleep.

### 2.1. Perceptual Measures

#### 2.1.1. Sleepiness

**Figure 2:**
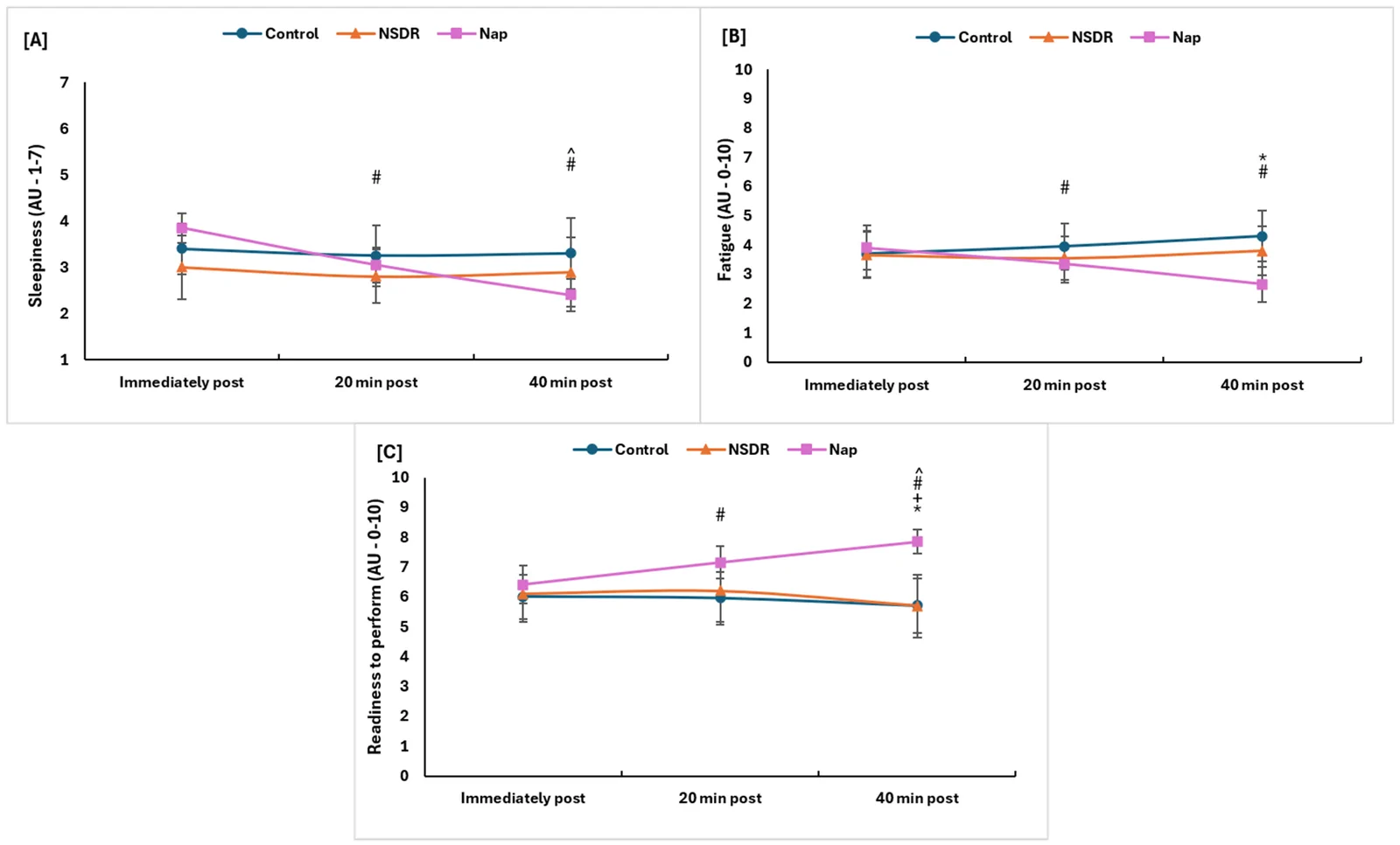
Comparison of sleepiness (**A**), fatigue (**B**), and readiness to perform (**C**) scores between nap, non-sleep deep rest (NSDR), and control groups at post, 20-min, and 40-min assessments. Data are presented as raw means with 95% confidence intervals. *: significant difference compared with control group; +: significant difference compared with NSDR group; #: significant difference compared with immediately post; ^: significant difference compared with 20-min post.

There was an interaction between Group and Time for sleepiness (F_(4, 109)_ = 4.83, *p* = 0.001; Table 2; Figure 2A). There were no significant between-group differences at any single timepoint (*p* > 0.05). Within the nap condition, sleepiness decreased from immediately post-intervention to 20 min post intervention (3.9 ± 0.7 to 3.1 ± 0.8 arbitrary units (AU), difference in estimated marginal means (ΔEMM) = 0.80 AU, 95% confidence interval (CI) [0.16, 1.44], *p* = 0.008) and from immediately post-intervention to 40 min post-intervention (3.9 ± 0.7 to 2.4 ± 0.8 AU, ΔEMM = 1.45 AU, 95% CI [0.81, 2.09], *p* < 0.001). Sleepiness was also lower at 40 min compared with 20 min post-intervention within the nap condition (2.4 ± 0.8 vs. 3.1 ± 0.8 AU, ΔEMM = 0.65 AU, 95% CI [0.01, 1.29], *p* = 0.04).

#### 2.1.2. Fatigue

A significant group × time interaction was observed for fatigue (F_(4, 109)_ = 8.74, *p* < 0.001; Table 2; Figure 2B). At 40 min post-intervention, the nap group reported significantly lower fatigue than the control group (ΔEMM = 1.59, 95% CI [0.25, 2.94], *p* = 0.01, Cohen’s d = 0.98). No differences were observed between NSDR and control groups (*p* > 0.05). Within the control condition, fatigue increased from immediately post-intervention to 40 min post-intervention (3.7 ± 1.7 to 4.3 ± 1.9 AU, ΔEMM = −0.79, 95% CI [−1.43, −0.15], *p* = 0.01). Within the nap condition, fatigue decreased from immediately post-intervention to 40 min post-intervention (3.9 ± 1.6 to 2.7 ± 1.3 AU, ΔEMM = 1.25, 95% CI [0.62, 1.88], *p* < 0.001) and from 20 min to 40 min post-intervention (3.4 ± 1.4 to 2.7 ± 1.3 AU, ΔEMM = 0.70, 95% CI [0.07, 1.33], *p* = 0.02).

#### 2.1.3. Readiness to Perform

A significant group × time interaction was observed for readiness to perform (F_(4, 112)_ = 9.16, *p* < 0.001; Table 2; Figure 2C). At 40 min post-intervention, the nap group reported significantly higher readiness to perform than both the control (ΔEMM = −2.33, 95% CI [−3.83, −0.85], *p* < 0.001, Cohen’s d = 1.48) and NSDR (ΔEMM = 2.21, 95% CI [0.72, 3.72], *p* = 0.001, Cohen’s d = 1.25) groups. No differences were observed between NSDR and control groups (*p* > 0.05). Within the nap condition, readiness to perform increased from immediately post-intervention to 20 min post-intervention (6.4 ± 1.4 to 7.2 ± 1.2 AU, ΔEMM = −0.75, 95% CI [−1.36, −0.14], *p* = 0.01) and from immediately post-intervention to 40 min post-intervention (6.4 ± 1.4 to 7.9 ± 0.9 AU, ΔEMM = −1.45, 95% CI [−2.06, −0.84], *p* < 0.001). Readiness to perform was also higher at 40 min compared with 20 min post-intervention within the nap condition (7.9 ± 0.9 vs. 7.2 ± 1.2 AU, ΔEMM = −0.70, 95% CI [−1.31, −0.09], *p* = 0.01).

### 2.2. Cognitive Performance

No significant group × time interaction was observed for reaction time (F_(4, 107)_ = 2.27, *p* = 0.06; Table 2), Simon effect in reaction time (F_(4, 105)_ = 0.12, *p* = 0.97; Table 2), and accuracy percentage during the Simon task (F_(4, 107)_ = 1.25, *p* = 0.29; Table 2). No significant main effect of group was observed for reaction time (F_(2, 52)_ = 0.60, *p* = 0.55), Simon effect in reaction time (F_(4, 105)_ = 1.75, *p* = 0.18), or accuracy percentage (F_(2, 52)_ = 0.52, *p* = 0.59; Table S1). However, a significant main effect of time was observed for reaction time (F_(2, 107)_ = 16.83, *p* < 0.001; Table S1).

### 2.3. Physical Performance

There was an interaction between Group and Time for handgrip strength (F_(4, 113)_ = 4.00, *p* = 0.004; Table 2). While groups did not differ at any single timepoint, the NSDR group showed small within-condition reductions from post-intervention to 40 min (ΔEMM = 2.61 kg, 95% CI [0.15, 5.08], *p* = 0.03).

## 3. Discussion

This study is, to our knowledge, the first to compare a 25-min nap opportunity with an NSDR protocol in physically active adults. Participants who completed the nap condition reported lower fatigue compared with those in the control condition and greater readiness to perform compared with both the control and NSDR conditions at 40 min post-intervention. However, the significant between-group findings were limited to subjective fatigue and readiness, with no significant between-group benefits observed for cognitive performance or handgrip strength. These findings suggest that a brief nap may promote higher perceived readiness and lower fatigue in the period following the intervention, although these benefits appear to emerge after a short delay, possibly reflecting the dissipation of sleep inertia. No significant between-group differences in favor of NSDR were detected across any of the measured outcomes. These non-significant findings should not be interpreted as evidence that NSDR is ineffective or equivalent to the control condition, as the absence of a statistically significant difference does not show the absence of an effect.

Perceptual responses showed that only the nap condition influenced subjective states. Participants in the nap group reported lower fatigue compared with the control group and greater readiness to perform compared with both the control and NSDR groups at 40 min post-intervention. These effects appeared after a short delay, which may be consistent with the gradual dissipation of sleep inertia typically observed within the first hour after waking [17]. In contrast, NSDR did not lead to significant changes in any perceptual measures, which differs from a previous study that reported improved fatigue, alertness, and mood following NSDR conditions [12]. While both interventions encourage relaxation, only the nap involved actual sleep, which likely activated physiological processes that support reductions in fatigue and enhanced readiness. The statistical indices support this interpretation. The high intraclass correlations for fatigue (ICC = 0.75) and readiness to perform (ICC = 0.81) indicate stable individual ranking across repeated measures, while the low marginal R^2^ values (0.09–0.15) show that group allocation explained only a small proportion of the variance. Together, these findings suggest that interindividual factors may have played a stronger role than intervention type in shaping perceptual responses to NSDR, whereas the nap produced distinct significant effects despite this variability.

Cognitive performance, assessed through reaction time, accuracy, and Simon effect in the Simon task, did not differ significantly between the nap, NSDR, and control conditions at any time point. These null findings suggest that neither intervention produced measurable effects on processing speed or response accuracy under the present study conditions, where participants reported around 7 h of sleep the previous night on average. The absence of significant differences aligns with the idea that both interventions primarily influence perceptual or subjective states rather than higher-order cognitive processing when baseline alertness is already sufficient. It is also possible that the short task duration and lack of prior fatigue limited the sensitivity to detect significant changes in executive performance. Another consideration is that cognitive performance tends to show high interindividual stability, making between-group effects more difficult to detect. This is reflected in the present model statistics, where reaction time and accuracy exhibited high intraclass correlations (ICC = 0.77 and 0.59, respectively) and low marginal R^2^ values (0.09–0.11), while the Simon effect showed a comparatively lower ICC (0.20) and similarly low marginal R^2^ (0.10), indicating that individual differences explained most of the variance while group allocation contributed little. Additionally, the 25-min nap opportunity may not have been long enough to include deeper stages of sleep such as slow-wave sleep or REM sleep, which are known to support memory consolidation, emotional regulation, and executive functioning [18,19]. As a result, the absence of these restorative stages could explain the lack of cognitive improvement following the nap condition. Similarly, NSDR, which does not involve actual sleep, is unlikely to activate the same neural mechanisms associated with cognitive enhancement. Together, these factors suggest that in participants who had slept around 7 h on average the night before testing, the cognitive benefits of a short nap or NSDR are minimal and may not extend beyond perceptual or self-reported outcomes.

Handgrip strength showed a significant group × time interaction, but post-hoc comparisons revealed no significant differences between the nap, NSDR, and control conditions at any time point. This suggests that while performance patterns varied slightly over time, these changes were not significantly influenced by the type of intervention. Reliability and model fit indices support this interpretation. Handgrip strength demonstrated excellent reliability (ICC = 0.86), and the mixed model explained nearly all variance (conditional R^2^ = 0.95), with a substantial proportion attributed to fixed effects (marginal R^2^ = 0.63). These values indicate consistent measurement and limited potential for acute enhancements in strength across conditions. This outcome aligns with previous findings showing minimal effects of short daytime naps on muscle force production [8]. Given the short duration and absence of prior fatigue, it is plausible that neither a 25-min nap nor an NSDR condition induces the neuromuscular processes necessary to enhance strength within such a brief post-intervention period. Sex was a significant covariate for handgrip strength, suggesting that observed variability was mainly attributable to sex-related differences in maximal force capacity rather than effects of the nap or NSDR.

The delayed effects observed after the nap may be consistent with the gradual dissipation of sleep inertia following awakening. Immediately after the nap, greater amounts of light sleep and longer total sleep time may have contributed to higher sleepiness, higher fatigue, and lower readiness to perform, consistent with sleep inertia, which can last up to an hour after waking [17]. By 40 min post-nap, these responses had shifted in line with the perceptual outcomes. The observed reduction in heart rate during the nap may indicate autonomic relaxation; however, it cannot be determined from the present study whether this contributed to lower fatigue and greater readiness to perform. Previous studies have shown that naps, even of short duration, promote vagal dominance and support autonomic regulation, which in turn reduces perceived exertion and enhances readiness [20,21]. Therefore, autonomic activity represents one possible explanation for the observed pattern.

Although NSDR did not produce significant group-level improvements in perceptual or performance outcomes, previous research has proposed autonomic modulation, including enhanced vagal activity and reduced cardiovascular strain, as a potential mechanism associated with relaxation-based interventions [22,23,24]. However, the present study was not designed to establish such a mechanistic pathway. While no measurable effects were observed in the present study, NSDR may still facilitate a state of physiological relaxation that could be beneficial in applied settings.

A key distinction between the interventions is that the nap opportunity involved actual sleep, whereas NSDR involved a wakeful state of guided relaxation. Although sleep-dependent and autonomic mechanisms may contribute differently to the effects of these interventions, the present study cannot determine the physiological pathways underlying either intervention. Accordingly, the physiological findings should be interpreted descriptively rather than as evidence of mechanisms explaining the observed perceptual responses.

Some limitations should be acknowledged. The absence of true pre-intervention baseline measures for the main outcomes is an important limitation. A post-only design was intentionally adopted to minimize the potential influence of repeated testing before relaxation-based interventions such as NSDR and napping, where arousal, fatigue, and practice effects may occur. While this approach reduces internal control, it offers a more ecologically valid representation of how these interventions would be implemented in applied settings. However, as this was a parallel-group study, each condition included different participants, and it is possible that some between-group differences reflected pre-existing variation in sleep pressure, fatigue, sleepiness, or readiness rather than the intervention alone. Baseline comparisons indicated that the nap group was significantly older and shorter in height than the other groups, and these characteristics were not included as covariates in the main analyses. Randomization was used to minimize systematic baseline differences, and the analyses were adjusted for sex, previous night’s sleep duration, and weekly physical activity. However, these covariates cannot fully account for individual differences in baseline perceptual or performance states, nor for the observed age and height differences between groups. Therefore, the findings should be interpreted as post-intervention between-group differences, rather than direct evidence of change from pre- to post-intervention. Future studies should consider a crossover design or include baseline outcome measures while carefully managing potential arousal, fatigue, and practice effects from repeated testing. Habitual sleep duration, chronotype, habitual napping status, time since awakening, and sleep across multiple preceding nights were not assessed. These factors may have influenced nap propensity and post-nap responses and should therefore be considered in future studies. In addition, the lack of electroencephalography (EEG) data precluded a direct comparison of neural activity between nap and NSDR, limiting our ability to delineate mechanistic pathways with greater precision. Although the intervention durations were selected to reflect the practical use of each strategy, the nap condition involved a 25-min rest opportunity, whereas the NSDR and control conditions lasted 10 min. This difference limits the ability to determine whether the observed responses were due to sleep itself, the longer rest opportunity, or a combination of both. Future studies should consider including duration-matched conditions to better isolate the contribution of sleep from rest duration. The conditions also differed in posture and physical setup, with participants lying on mats during NSDR, sitting during the control condition, and using a sleeping pod during the nap condition. Although ambient temperature and light exposure were kept similar across conditions, these differences in posture and resting environment may have independently influenced subjective and autonomic responses. Future studies should consider posture- and environment-matched comparison conditions where feasible. In addition, physiological measures were not collected consistently across all conditions. Heart rate variability (HRV) and peripheral skin temperature were recorded only during the NSDR and control conditions, while heart rate was recorded using different devices during the nap condition and the NSDR/control conditions. This limits direct comparison of physiological responses between interventions. No mediation analysis was conducted; therefore, it cannot be determined whether physiological responses explained the observed reductions in fatigue or greater readiness to perform. Sleep during the NSDR condition was also not objectively monitored; therefore, brief episodes of sleep cannot be definitively excluded. Furthermore, as the sample comprised physically active young adults, the findings may not generalize to other populations, such as older adults, clinical populations, or individuals with lower habitual physical activity levels, and future research should examine whether these effects extend to broader populations.

## 4. Materials and Methods

### 4.1. Participants

G*Power software (Version 3.1.9.2; Kiel University, Kiel, Germany) [25] was used to calculate the required sample size. The α level was set at 0.05 and statistical power at 0.95. Effect size (Cohen’s f) was derived from the partial eta-squared value reported for handgrip strength in our previous NSDR study [12]. Handgrip strength was used as the primary outcome for the sample-size calculation. The estimated effect size was f = 0.50, requiring a minimum sample size of 18 participants to allow equal allocation across the three groups. A total of 60 participants were recruited, with 20 participants allocated to each condition, to account for potential drop-out and ensure that statistical power remained above 95%.

Sixty physically active young adults (26 female, 34 male; mean age 22 ± 4 years) participated in the current study. Participants were randomly allocated to one of three conditions: nap, NSDR, or control (*n* = 20 per group). Physical activity was assessed using a single self-reported item asking participants to report the number of hours of physical activity completed in a typical week. On average, participants reported engaging in 8 ± 3 h of physical activity per week across a variety of sports (e.g., Australian football, soccer, rugby, basketball). All participants were free from musculoskeletal injuries and self-reported sleep or medical disorders, and were not using medications known to affect sleep, cognition, or physical performance. Written informed consent was obtained from all participants prior to data collection. The study was conducted in accordance with the Declaration of Helsinki and approved by the Human Research Ethics Committee at La Trobe University (approval number: HEC23255).

### 4.2. Experimental Design

The study employed a randomized, parallel-group design with three experimental arms: a 25-min nap opportunity, a 10-min non-sleep deep rest condition (NSDR), and a no-intervention control. Participants were randomly allocated to one of the three groups, and assessments were conducted at three time points: immediately, 20 min, and 40 min post-intervention. Randomization was performed by preparing identical papers labeled with each group, which were mixed and distributed to participants. Each participant opened their assigned paper to reveal their group. Participants were instructed to refrain from caffeine, alcohol, and vigorous exercise for at least 4 h before testing. We employed a post-only assessment schedule following all three conditions to characterize the time-course of performance readiness while minimizing pre-test contamination. Same-session pre-testing was avoided because it can elevate arousal, induce fatigue, and introduce practice effects that may attenuate intervention effects for relaxation-based protocols (nap/NSDR). The 0–40 min window was chosen to capture recovery from any transient sleep inertia after the 25-min nap and to align with real-world use cases (e.g., return-to-play intervals).

The intervention durations were selected to reflect the practical format of each strategy. The 10-min NSDR duration was chosen because NSDR is commonly delivered as a brief guided relaxation protocol and may be most useful in settings where time available for rest is limited. The 25-min nap opportunity was selected to allow sufficient time for sleep onset and a short period of sleep, while limiting the likelihood of prolonged sleep inertia. This duration was also informed by previous work showing that a 25-min nap opportunity can result in approximately 10 min of actual sleep [26].

The intervention period for all three conditions was conducted under similar ambient room conditions, with room temperature maintained at 21–23 °C and light exposure kept below 5 lux.

**Figure 3:**
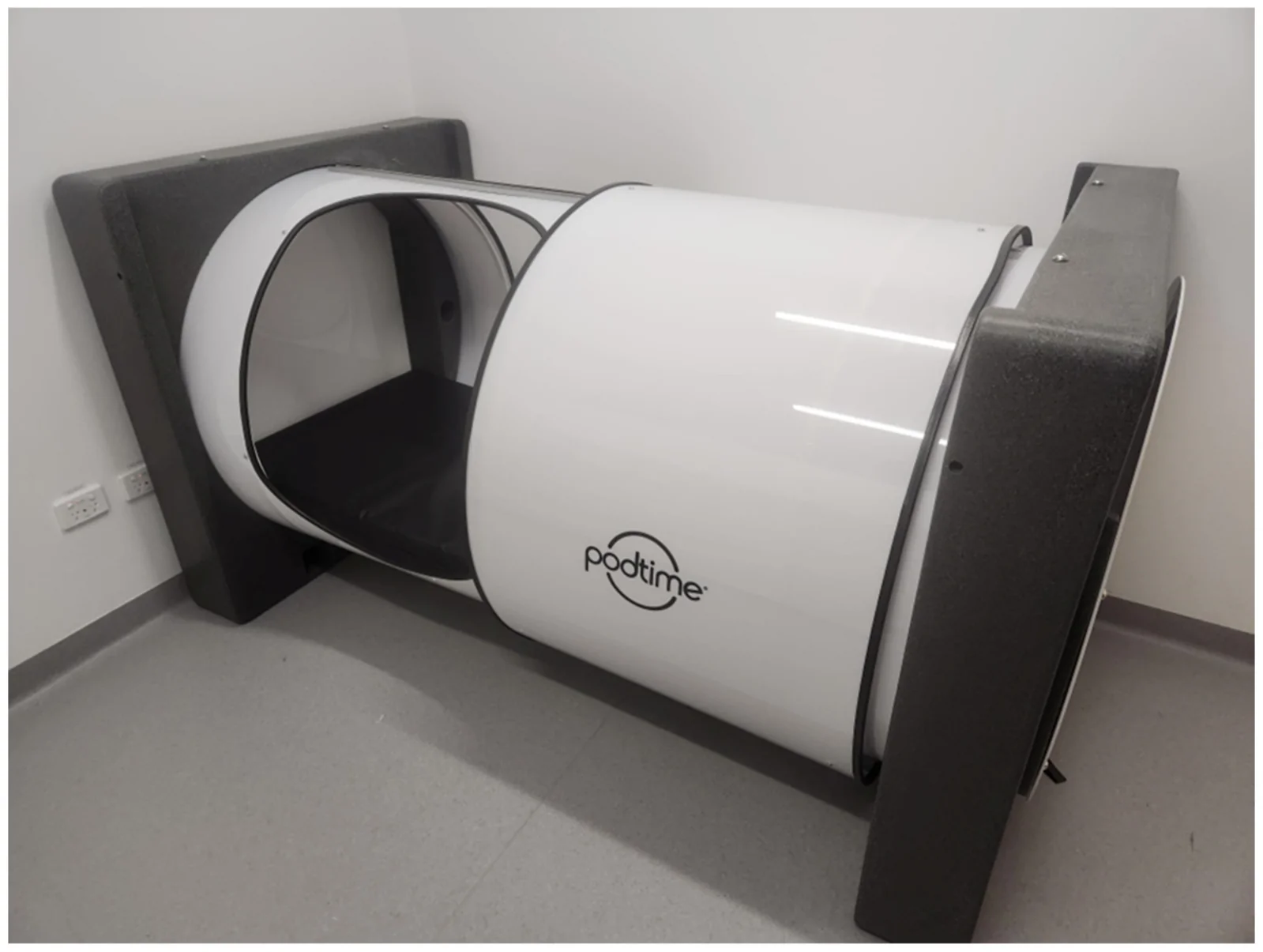
A photo of the napping pod where the 25-min nap opportunity took place.

For the nap condition, the session was performed in a quiet sleep room equipped with a napping pod (Podtime, Restworks, Perth, Australia) (Figure 3). Participants in the nap condition were included in the analysis only if sleep was obtained during the 25-min nap opportunity. The nap opportunity took place between 14:35 h and 15:00 h, and sleep was monitored using the Somfit wearable EEG device (Compumedics Ltd., Melbourne, Australia).

For the NSDR and control conditions, testing was conducted at 15:00 h. The NSDR group completed a 10-min guided session delivered through a standardized audio protocol (https://youtu.be/AKGrmY8OSHM, accessed on 26 August 2026), lying comfortably on mats in a quiet room. Participants were instructed to remain awake and follow the standardized audio protocol throughout the 10-min session. However, adherence to the audio instructions was not formally assessed, and sleep during NSDR was not objectively monitored using EEG. In the control condition, participants sat quietly in the same environment under supervision, without engaging in relaxation exercises, sleep, or the use of electronic devices. Physiological responses (heart rate (HR), HRV, and skin temperature) were continuously recorded in both NSDR and control using a smart ring (Ultrahuman Ring AIR, Ultrahuman Healthcare, Bangalore, India).

Following the intervention period, participants completed perceptual (sleepiness, fatigue, readiness to perform), cognitive (Simon task), and physical (handgrip strength) assessments at each of the three post-intervention time points.

### 4.3. Sleep Monitoring During Naps

Sleep during the nap opportunity was assessed using a wearable EEG device (Somfit, Compumedics, Melbourne, Australia). The Somfit is a portable system that attaches to the forehead with an adhesive patch and transmits data to a companion mobile application (iOS/Android). The Somfit records single-channel EEG (Fp1–Fp2), electro-oculography (Fp1–Fpz, Fp2–Fpz), electromyography (Fp1–Fp2), pulse oximetry, head position, snoring sound, movement, and ambient light. Validation against polysomnography (PSG) has shown an overall agreement of 76.1% between Somfit’s automated scoring and consensus PSG hypnograms, with a Cohen’s kappa of 0.67. Sleep/wake classification accuracy was 89.3%, comparable to inter-scorer variability observed among PSG technologists [27].

### 4.4. Physiological Monitoring During NSDR and Control Conditions

Physiological responses during the NSDR and control conditions were continuously recorded using a finger-worn smart ring device (Ultrahuman Ring Air, Ultrahuman Healthcare, Bangalore, India). The ring integrates a photoplethysmography (PPG) sensor to capture heart rate and heart rate variability (HRV, expressed as the root mean square of successive differences (RMSSD); other HRV metrics such as the standard deviation of normal-to-normal intervals (SDNN), high frequency (HF), or low frequency (LF) were not available from this device), along with a non-contact sensor to monitor peripheral skin temperature. For each 10-min intervention, start and end values of HR, HRV, and temperature were extracted, as well as average values across the session (for HRV and temperature) and the minimum HR reached during the period.

### 4.5. Perceptual Measures

Subjective sleepiness was assessed using the Stanford Sleepiness Scale, where participants rated their current level of alertness on a 7-point scale ranging from 1 (“active, alert, fully awake”) to 7 (“near sleep onset, struggling to remain awake”). This tool has shown good validity and reliability, with 88% agreement in prior assessments [28].

Whole-body fatigue was measured on a 10-point numerical scale [29], with 0 representing “normal, without pain or stiffness” and 10 reflecting “extreme fatigue.”

Readiness to perform was evaluated using a comparable 10-point scale [29], in response to the question, “How ready are you to perform?” where 0 indicated “not at all ready” and 10 indicated “extremely ready.”

### 4.6. Cognitive Performance

Cognitive control was assessed using the Simon task, a well-established measure of interference control and response inhibition [30]. Participants were seated at a laptop and completed the task through an open-source software platform (The Psychology Experiment Building Language, PEBL, Version 2.1) [31]. They were instructed to respond as quickly and accurately as possible to the color of a circle (red or blue) that appeared to the left or right of a central fixation point, while ignoring its spatial location. Responses were made using the thumbs: the left shift key for red and the right shift key for blue.

Before the experimental trials, participants completed a familiarization session to ensure they understood the task instructions and which key to press for each stimulus color.

The task consisted of 70 randomly ordered trials, with equal proportions of congruent trials (stimulus location matched response side) and incongruent trials (stimulus location opposite to response side). Each stimulus remained on screen until a response was given or for a maximum of 1.5 s, after which the next trial appeared. Two performance outcomes were calculated: mean reaction time for correct responses, reflecting processing speed, and percentage of correct responses, reflecting accuracy. The Simon effect, reflecting interference control, was calculated as the difference in mean reaction time between incongruent and congruent trials.

### 4.7. Physical Performance

Handgrip strength was selected as the physical performance outcome because it provides a brief, objective, and reliable measure of maximal force that can be repeatedly assessed within a short post-intervention period. Importantly, handgrip strength also showed an acute improvement following the same NSDR protocol in our previous study [12], providing a specific rationale for examining whether this response was reproduced when NSDR was compared with napping and quiet rest. Maximal grip strength of the dominant hand was assessed using a Jamar Plus Digital hand dynamometer (Paterson Medical, Green Bay, WI, USA). The device was individually adjusted to accommodate hand size, and measurements were taken in a standardized position: participants stood upright with shoulders adducted and neutrally rotated, elbows flexed at 90°, forearms in neutral alignment, wrists extended between 0–30° with 0–15° ulnar deviation, and feet flat on the floor. Each participant performed three maximal-effort trials, separated by 30 s of passive rest, and the highest value (kg) was used for analysis. This protocol is considered the gold standard for grip strength assessment and has demonstrated excellent validity and reliability [32].

### 4.8. Statistical Analysis

All data were analyzed using R (version 4.5.1; R Core Team, Vienna, Austria). Linear mixed-effects models (LMMs) were used to examine the effects of group (Nap, NSDR, Control), time (immediately post, 20-min post, 40-min post), and their interaction on perceptual (sleepiness, fatigue, readiness to perform), cognitive (reaction time, accuracy, and the Simon effect in reaction during the Simon task), and physical outcomes (handgrip strength). Participant ID was included as a random effect to account for repeated measures. Sex, self-reported sleep duration, and weekly physical activity were entered as covariates in all models.

Baseline group characteristics (age, body mass, height, physical activity, and previous night’s sleep duration) were compared across the three groups using one-way analysis of variance (ANOVA). The distribution of sex across groups was compared using a chi-square test.

Model fit and variance components were quantified using intra-class correlation coefficients (ICC), marginal R^2^ (variance explained by fixed effects), and conditional R^2^ (variance explained by fixed + random effects).

When significant interaction effects were detected, pairwise post-hoc comparisons were performed using estimated marginal means with Bonferroni correction to adjust for multiple testing. Effect sizes were expressed as partial eta-squared (η_p_^2^) with values interpreted as: small (0.01), medium (0.06), and large (0.14) [33].

The significance level was set at *p* < 0.05. Exact *p*-values are reported; those below 0.001 are denoted as *p* < 0.001. Descriptive values presented in the tables and figures are reported as mean ± standard deviation (SD).

## 5. Conclusions

A 25-min nap opportunity and a 10-min NSDR condition produced distinct patterns of response. The nap condition led to reductions in fatigue compared with the control condition and greater readiness to perform compared with both the control and NSDR conditions at 40-min post-intervention. These delayed effects likely reflect the dissipation of sleep inertia and the autonomic adjustments that accompany light sleep (N1 and N2), which together contribute to improved perceptual states. In contrast, no significant post-intervention between-group differences were observed in favor of NSDR across perceptual, cognitive, or physical measures. These non-significant findings should not be interpreted as evidence that NSDR is ineffective or equivalent to the control condition. Overall, these results indicate that short naps can enhance perceived readiness and reduce fatigue when sufficient time is allowed post-awakening, whereas NSDR may still hold potential as a relaxation strategy; however, under the present conditions, it was not associated with post-intervention outcomes that differed significantly from the control condition.

## Figures and Tables

**Table 1:** Participant characteristics in the control, non-sleep deep rest (NSDR), and nap groups.

	Control Group	NSDR Group	Nap Group	*p* Value
**Female participants (*n*)**	7	9	10	0.62
**Male participants (*n*)**	13	11	10	
**Age (years)**	21 ± 2	21 ± 3	25 ± 4	0.003
**Body Mass (kg)**	74 ± 11	72 ± 14	66 ± 10	0.11
**Height (cm)**	176 ± 10	173 ± 10	168 ± 10	0.04
**Physical activity (hours/week)**	9 ± 3	9 ± 3	7 ± 2	0.07
**Previous night’s sleep duration (** **h** **:min)**	7:09 ± 1:12	6:33 ± 1:06	7:10 ± 1:00	0.14

**Table 2:** Comparison of cognitive and physical measures between nap, non-sleep deep rest (NSDR) and control groups at post, 20-min, and 40-min assessments. Bold values represent significant group × time interactions (*p* < 0.05).

	Control			NSDR			Nap			Group × Time	
	IP	P20	P40	IP	P20	P40	IP	P20	P40	*p* Value	Effect Size (η_p_^2^)
**Reaction time (ms) $**	434 ± 48	411 ± 47	405 ± 43	409 ± 45	397 ± 46	402 ± 39	433 ± 63	409 ± 47	400 ± 41	0.06	0.08
**Accuracy (%)**	93 ± 3	93 ± 4	93 ± 4	94 ± 4	93 ± 5	95 ± 5	94 ± 4	95 ± 3	96 ± 2	0.29	0.04
**Simon effect in reaction time (ms)**	46 ± 32	48 ± 41	52 ± 59	36 ± 23	37 ± 31	45 ± 47	26 ± 32	27 ± 29	37 ± 24	0.97	0.005
**Handgrip strength (kg)**	44 ± 15	42 ± 14	42 ± 13	43 ± 15	41 ± 13	40 ± 13 #	36 ± 11	37 ± 11	38 ± 11	**0.004**	0.13

$: reaction time measures taken from correct trials during the Simon task; #: significant difference compared with immediately post; IP = immediately post-intervention; P20 = 20 min post-intervention; P40 = 40 min post-intervention.

## Data Availability

The data that support the findings of this study are available from the corresponding author upon reasonable request.

## Supplementary Materials

The following supporting information can be downloaded at: https://www.mdpi.com/article/10.3390/clockssleep8030052/s1, Table S1: Linear mixed-effects models results for perceptual, cognitive, and physical measures; Table S2: Intra-class correlation coefficients (ICC), marginal R^2^, and conditional R^2^ for perceptual, cognitive, and physical measures; Table S3. Physiological measures (heart rate (HR), heart rate variability (HRV), and skin temperature) during the 25-min nap opportunity, NSDR and control conditions.
