# Supplementary material for: Comparing the Effects of a Short Nap and Non-Sleep Deep Rest on Perceptual, Cognitive, and Physical Performance in Active Adults
Source: Clocks Sleep. 2026 Aug 28;8(3):52. doi: 10.3390/clockssleep8030052 (PMC13605941; doi:10.3390/clockssleep8030052)
Supplement: Supplementary file 1 [file clockssleep-08-00052-s001.zip › clockssleep-4435273-supplementary.pdf]

Table S1. Linear mixed-effects models results for perceptual, cognitive, and physical measures.

	Fixed effect	F	p	Effect size (η_p^2)
**Sleepiness**	Group	0.36	0.69	0.01
	Time	5.71	0.004	0.09
	Group × Time	4.83	0.001	0.15
	Sex	0.02	0.88	0.0004
	Sleep duration	0.37	0.54	0.007
	Physical activity	0.71	0.40	0.01
**Fatigue**	Group	0.53	0.59	0.02
	Time	0.21	0.81	0.003
	Group × Time	8.74	< 0.001	0.24
	Sex	0.73	0.39	0.01
	Sleep duration	0.13	0.71	0.002
	Physical activity	0.91	0.34	0.02
**Readiness to perform**	Group	3.54	0.03	0.12
	Time	1.66	0.19	0.03
	Group × Time	9.16	< 0.001	0.25
	Sex	1.17	0.28	0.02
	Sleep duration	0.04	0.83	0.0008
	Physical activity	0.01	0.90	0.0002
**Reaction time for correct trials during Simon task**	Group	0.60	0.55	0.02
	Time	16.83	< 0.001	0.21
	Group × Time	2.27	0.06	0.08
	Sex	0.07	0.79	0.003
	Sleep duration	0.27	0.60	0.005
	Physical activity	0.16	0.69	0.003
**Accuracy percentage during Simon task**	Group	0.52	0.59	0.02
	Time	2.53	0.08	0.05
	Group × Time	1.25	0.29	0.04
	Sex	0.07	0.79	0.003

	Sleep duration	0.34	0.55	0.006
	Physical activity	3.35	0.07	0.06
**Simon effect in reaction time**	Group	1.75	0.18	0.07
	Time	1.05	0.35	0.02
	Group × Time	0.12	0.97	0.005
	Sex	0.29	0.58	0.006
	Sleep duration	3.22	0.07	0.06
	Physical activity	1.33	0.25	0.03
**Handgrip strength**	Group	0.33	0.71	0.01
	Time	1.62	0.20	0.03
	Group × Time	4.00	0.004	0.13
	Sex	77.89	< 0.001	0.60
	Sleep duration	0.15	0.69	0.002
	Physical activity	3.80	0.05	0.07

Table S2. Intra-class correlation coefficients (ICC), marginal R^2, and conditional R^2 for perceptual, cognitive, and physical measures.

	Adjusted ICC	Conditional R^2	Marginal R^2
**Sleepiness**	0.581	0.622	0.099
**Fatigue**	0.752	0.773	0.086
**Readiness to perform**	0.809	0.837	0.145
**Mean reaction time during Simon task**	0.773	0.792	0.085
**Accuracy during Simon task**	0.588	0.631	0.105
**Simon effect in reaction time**	0.196	0.274	0.097
**Handgrip strength**	0.856	0.946	0.625

Table S3. physiological measures (heart rate (HR), heart rate variability (HRV), and skin temperature) during the 25-min nap opportunity, NSDR and control conditions.

**25-min nap opportunity**	Average HR (bpm)	72 ± 9
	Lowest HR (bpm)	63 ± 11
	Highest HR (bpm)	83 ± 10
**NSDR condition**	HR at the start session (bpm)	89 ± 17
	HR at the end session (bpm)	82 ± 21
	Lowest HR (bpm)	66 ± 8
	HRV at the start session (ms)	96 ± 57
	HRV at the end session (ms)	68 ± 45
	Average HRV (ms)	64 ± 32
	Skin temperature at the start session (°C)	30 ± 4
	Skin temperature at the end session (°C)	33 ± 4
	Average temperature (°C)	32 ± 4
**Control condition**	HR at the start session (bpm)	89 ± 33
	HR at the end session (bpm)	82 ± 17
	Lowest HR (bpm)	69 ± 8
	HRV at the start session (ms)	65 ± 41
	HRV at the end session (ms)	50 ± 27
	Average HRV (ms)	57 ± 28
	Temperature at the start session (°C)	29 ± 3
	Temperature at the end session (°C)	33 ± 3
	Average temperature (°C)	32 ± 3

Values are presented descriptively only. Physiological measures were not collected consistently across all intervention conditions and were not used for direct statistical comparison between interventions.
